# Using web-based, guided self-help to bridge the waiting time for face-to-face out-patient treatment for bulimic-spectrum disorders: randomised controlled trial

**DOI:** 10.1192/bjo.2023.629

**Published:** 2024-02-26

**Authors:** Bianka Vollert, See Heng Yim, Dennis Görlich, Ina Beintner, Gemma Gordon, Peter Musiat, Ulrike Schmidt, Corinna Jacobi

**Affiliations:** Institute of Clinical Psychology and Psychotherapy, Technische Universität Dresden, Germany; Institute of Psychiatry, Psychology and Neuroscience, Department of Psychological Medicine, King's College London, UK; Institute of Biostatistics and Clinical Research, Westfälische Wilhelms-Universität Münster, Germany; Institute of Psychiatry, Psychology and Neuroscience, Department of Psychological Medicine, King's College London, UK; and Forward College, Lisbon, Portugal; Institute of Psychiatry, Psychology and Neuroscience, Department of Psychological Medicine, King's College London, UK; and South London and Maudsley NHS Foundation Trust, London, UK

**Keywords:** Eating disorders, web-based interventions, guided self-help, out-patients, randomised controlled trial

## Abstract

**Background:**

Although effective treatments for bulimic-spectrum eating disorders exist, access is often delayed because of limited therapist availability and lengthy waiting lists. Web-based self-help interventions have the potential to bridge waiting times for face-to-face treatment and overcome existing treatment gaps.

**Aims:**

This study aims to assess the effectiveness of a web-based guided self-help intervention (everyBody Plus) for patients with bulimia nervosa, binge eating disorder and other specified feeding and eating disorders who are waiting for out-patient treatment.

**Method:**

A randomised controlled trial was conducted in Germany and the UK. A total of 343 patients were randomly assigned to the intervention ‘everyBody Plus’ or a waitlist control condition. The primary outcome was the number of weeks after randomisation until a patient achieved a clinically relevant improvement in core symptoms for the first time. Secondary outcomes included eating disorder attitudes and behaviours, and general psychopathology.

**Results:**

At 6- and 12-month follow-up, the probability of being abstinent from core symptoms was significantly larger for the intervention group compared with the control group (hazard ratio: 1.997, 95% CI 1.09–3.65; *P* = 0.0249). The intervention group also showed larger improvements in eating disorder attitudes and behaviours, general psychopathology, anxiety, depression and quality of life, compared with the control group at most assessment points. Working alliance ratings with the online therapist were high.

**Conclusions:**

The self-help intervention everyBody Plus, delivered with relatively standardised online guidance, can help bridge treatment gaps for patients with bulimic-spectrum eating disorders, and achieve faster and greater reductions in core symptoms.

Bulimic-spectrum eating disorders, i.e. bulimia nervosa, binge eating disorder (BED) and other specified feeding or eating disorders with symptoms of binge eating (OSFED), are common, disabling and associated with high disease burden.^1^ International guidelines recommend guided self-help interventions as first-line treatment for bulimic-spectrum eating disorders because of their proven efficacy and because they represent an efficient and cost-effective use of limited therapist resources.^2–4^ Eating disorder services often have lengthy waiting lists for treatment, with patients with bulimic-spectrum disorders being deprioritised compared with those with anorexia nervosa. The problem of lengthy waiting lists has recently been compounded by the rising referrals to eating disorder services internationally since the COVID-19 pandemic.^5^ Web-based guided self-help has shown promise in reducing eating disorder symptoms, as well as full and subthreshold eating disorders.^3,6,7^ Moreover, individuals with eating disorders and their carers consider it a useful way to reduce barriers to accessing care for this group.^8^ Through increasing access, this could reduce the duration of untreated eating disorders, increasing the likelihood of remission^9^ and reducing drop-out during subsequent treatment.^10^ We therefore aimed to evaluate the effectiveness of a web-based guided self-help programme (everyBody Plus) for women seeking out-patient treatment for bulimic-spectrum disorders in a pragmatic, randomised controlled trial (RCT) in Germany and the UK. Our overall goal was to reduce the treatment gap by bridging the waiting time for face-to-face out-patient interventions. To the best of our knowledge, this is the first large-scale trial trying to address this gap.^11^ Our main hypothesis was that women offered everyBody Plus before starting usual face-to-face therapy would achieve clinically relevant symptom improvement quicker than those who have to wait for treatment.

## Method

### Study design

The study was a pragmatic RCT comparing everyBody Plus (intervention group) and a waiting list control group. Assessments took place at pre-intervention, mid-intervention (4 weeks after randomisation), post-intervention, and 6- and 12-month follow-up. A weekly symptom diary was used to collect data on core eating disorder symptoms. Because of the pragmatic nature of the trial, patients could start their regular therapy irrespective of their progress in the study and intervention completion. The authors assert that all procedures contributing to this work comply with the ethical standards of the relevant national and institutional committees on human experimentation and with the Helsinki Declaration of 1975, as revised in 2008. Ethical approval was sought (ethics committee of Technische Universität Dresden in Germany: approval number EK 84032016; Health Research Authority in the UK: approval number 16/NW/0888). Participants gave electronic consent by ticking a checkbox on the study platform. By ticking the box, they confirmed that they had thoroughly read and understood the information about the study procedures and data security measures provided to them. This approach is compliant with the European Union's General Data Protection Regulation and was approved by the ethics committee. The trial is registered within the ISRCTN registry (identifier ISRCTN12608780). The trial protocol is available elsewhere.^11^

#### Intervention

everyBody Plus is a web-based, cognitive–behavioural guided self-help intervention based on the well-researched ‘StudentBodies’ programme for females with subclinical eating disorders.^12^ The current intervention was designed for adult women with bulimia nervosa, BED or OSFED with clinical-level binge eating who were awaiting face-to-face treatment. Eight modules (i.e. 8 weekly sessions) covered a range of eating disorder-related topics, such as balanced eating, exercise patterns, dealing with ‘forbidden foods’, binge eating/purging, improving body image, dealing with emotions, perfectionism and self-esteem. The programme included interactive elements such as weekly symptom monitoring diaries addressing body weight, frequency of binge eating and compensatory behaviours in the past week; self-reflection diaries; free-text responses within each session; group forums and homework tasks. Each session took about 1 h to complete. The intervention is described in more detail in the study protocol.^11^

Patients allocated to everyBody Plus received weekly, individualised feedback based on their diary entries and free-text responses, irrespective of their engagement with the intervention. The feedback addressed topics such as motivation, encouraging reflection and participation, clarification and signposting. Patients without any activity in the programme in the past week received motivational messages. In Germany, participant feedback was provided by B.V. under supervision from I.B. In the UK, participant feedback was mainly provided by S.H.Y. Twelve other trained eating disorder psychological therapists and psychology postgraduates took part as ‘online therapists’, under the supervision of qualified psychological therapists. Typically, online therapists took 20–30 min to complete a weekly feedback message.

#### Control condition

Patients allocated to the waiting list control condition were prompted to complete the symptom diary and other measures. No therapist feedback was provided.

### Participants and procedure

In Germany, patients were recruited via 72 clinical therapists in private practices and 24 out-patient treatment centres. In addition, a self-referral strategy through counselling centres (partially specialised in eating disorders), social media and newspaper articles was implemented. In this case, a licensed physician or psychologist had to confirm the diagnosis, and study eligibility was checked by the study team at baseline assessment. In the UK, patients were recruited through 15 National Health Service (NHS) Foundation Trusts, national and regional eating disorder charities, King's College London email circulars and self-referral. Eligibility of the patients recruited through the NHS was screened by the respective eating disorders team, and those recruited through other sources were screened against DSM-5 criteria by the research team at King's College London.

Female patients aged 18 years or older were included if they met diagnostic criteria, based on the DSM-5,^13^ for bulimia nervosa, BED or OSFED with binge eating; if they were currently on the waiting list for psychological interventions or seeking treatment; if their English or German language proficiency was sufficient to participate in the study and if they had access to the internet. Exclusion criteria were a body mass index (BMI) score <18.5 kg/m^2^, requiring day patient or in-patient eating disorder treatment, significant psychiatric or medical comorbidity or active suicidality, or receiving antidepressant medication on a stable dose for <4 weeks.

Following baseline assessment, eligible patients were randomised in a 1:1 ratio to the two study arms, stratified by country (Germany, UK). A block randomisation with fixed block size was used to generate randomisation lists per stratum. Randomisation lists were generated by Westfälische Wilhelms-UniversitätMünster, which was independent from the participant enrolment (Technische Universität Dresden, King's College London). A central web-based randomisation service managed by Westfälische Wilhelms-UniversitätMünster was used to maintain allocation concealment.

All assessments were collected online.

### Primary outcome

The primary outcome was the number of weeks after randomisation until a patient achieved a clinically relevant improvement in core eating disorder symptoms, based on the weekly symptom diary. This was defined as abstinence from binge eating and compensatory behaviours (vomiting, fasting; use of laxatives, diuretics and/or appetite suppressants) and a BMI of ≥18.5 kg/m^2^ over a period of at least 4 consecutive weeks. The occurrence of a clinically relevant improvement according to the definition was considered as an event. To assess the primary outcome, patients were asked to monitor their eating disorder symptoms (binge eating and compensatory behaviours) and body weight over a period of 1 year, and reminded to provide frequencies as numeric variables in the diary on a weekly basis. If the frequency of binge eating episodes and all compensatory behaviours was 0, a patient was considered as abstinent in the respective week the diary had been filled in. The date of the fourth diary entry without any binge eating or compensatory behaviours in combination with a BMI of ≥18.5 kg/m^2^ was used to calculate the number of weeks after randomisation that the event occurred for the first time.

### Secondary outcomes

Secondary outcomes were core eating disorder symptoms and attitudes, measured with the Eating Disorder Examination Questionnaire (EDE-Q),^14^ the Weight Concerns Scale (WCS)^15^ and the Intuitive Eating Scale (IES),^16^ and described in detail in the study protocol.^11^

Eating disorder diagnoses were based on EDE-Q diagnostic items.^14^ Bulimia nervosa was defined as having objective binge eating episodes and vomiting as means of weight control at least four times in the past 28 days. BED was defined as eating large amounts of food at least four times in the past 28 days without vomiting. All other patients in the trial were classified as OSFED.

Self-reported weight and height were used for BMI calculation. Other measures covered general psychopathology, including depression (Patient Health Questionnaire-9 (PHQ-9)^17^), anxiety (Generalised Anxiety Disorder-7 (GAD-7)^18^), alcohol consumption (Alcohol Use Disorders Identification Test-Consumption (AUDIT-C)^19^), self-esteem (Rosenberg Self-Esteem Scale (RSE)^20^) and quality of life (Assessment of Quality of Life-8D (AQoL-8D)^21^).

Process measures included the Working Alliance Inventory – Short Revised (WAI-SR).^22^ Patients were also asked to complete a satisfaction rating after each session. Adherence to the intervention was operationalised as the number of completed sessions, diary entries and messages written in the discussion board or to the online coach.

### Statistical analysis

#### Primary confirmatory analysis

The effect of the programme was tested between the two study arms by log-rank tests on the primary outcome, applying the intention-to-treat (ITT) principle. The primary analysis was performed on a multiple significance level of 5% within a two-stage flexible design, according to a group sequential plan,^23^ with one interim analysis. The interim analysis was planned after 39 patients with improvements had been observed. The final analysis was planned after 77 ‘events’.

Data were continuously monitored to detect improvements in core eating disorder symptoms. Although the recruitment aims were met because of a lower than predicted number of events, the interim analysis could not be triggered until shortly before study end, with 44 events. Accordingly, the interim and the final data-sets are identical. We therefore only report the results of the latter (*N* = 337).

#### Per-protocol analysis of the primary outcome

The per-protocol cohort contains data from all patients in the intervention group who opened the first session and logged on to the platform at least one more time, as well as the control group patients. This coincides with the ITT cohort for the time-to-event analysis with observation times >0. Because the ITT and per-protocol cohorts of the primary outcome are identical, we will not report an additional per-protocol analysis.

#### Secondary analyses

Primary and secondary outcomes were analysed with multilevel mixed effect models (MMEM) following the ITT principle. Group comparisons were computed at each assessment point by constructing the corresponding contrast from the MMEM. Missing data were handled using full information maximum likelihood estimation. Categorical variables were analysed with Fisher's exact test or chi-squared tests. Changes in core eating disorder symptoms at follow-up for assessment completers were analysed by calculating a change score with respect to the baseline situation (i.e. −1: stopped the behaviour, 0: no change; 1: onset). Change scores were tested by chi-squared test between study arms. Eating disorder core symptom frequencies (assessed over the past 28 days) were compared by Mann–Whitney *U*-tests. A significance level of 0.05 (two-tailed) for all secondary analyses was applied.

#### Effect sizes

For the primary outcome, a hazard ratio estimated by a Cox proportional hazard model was provided as effect size, to assess the difference in chance of experiencing symptom improvement between the intervention group and control group. Additionally, we calculated a number needed to treat following the method detailed by Altman and Andersen.^24^ For continuous secondary outcomes, Cohen's *d* was estimated from the linear mixed models (ITT analysis) based on the estimated (marginal) means per group and assessment in combination with the estimated pooled s.d.

## Results

### Participant characteristics

**Fig. 1:**
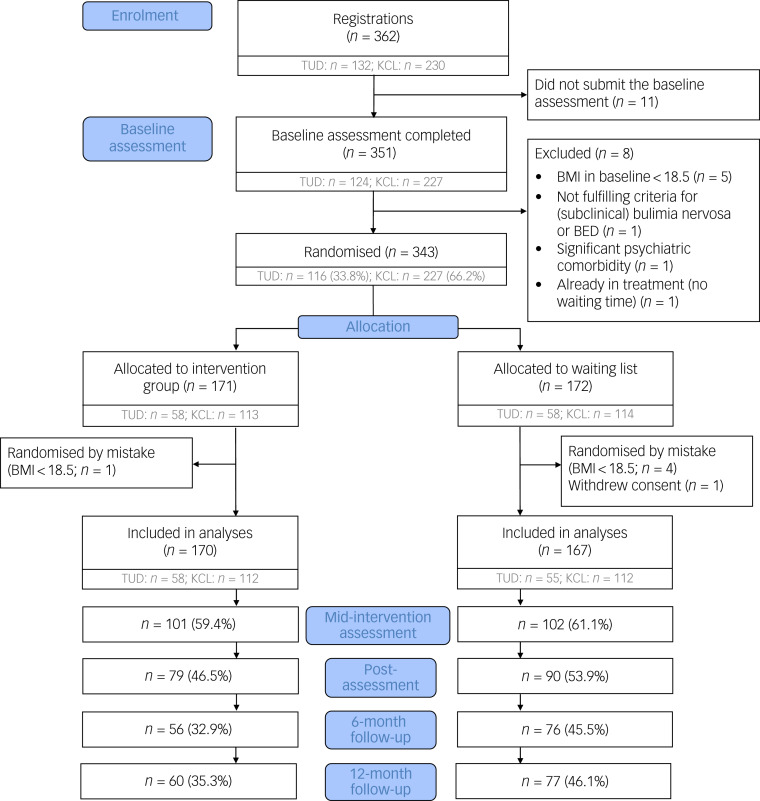
Participant flow chart. Because the numbers of completed questionnaires at each assessment point varied between patients, numbers in the Consolidated Standards of Reporting Trials (CONSORT) flow chart indicate patients with complete assessments at the respective time point. BED, binge eating disorder; BMI, body mass index; KCL, King's College London; TUD, Technische Universität Dresden.

Participants were enrolled between 29 November 2016 and 28 May 2019. A total of 362 patients registered for study participation, of which 343 were randomised. Five patients were randomised erroneously (BMI < 18.5 kg/m^2^) and one participant withdrew their consent after baseline. The final baseline sample size of the study was 337 (intervention group: *n* = 170; control group: *n* = 167; Fig. 1).

A third of the sample was recruited in Germany (*n* = 113) and two-thirds were recruited in the UK (*n* = 224). In Germany, 22 patients were recruited through out-patient therapy centres, 56 through therapists in private practice and 35 self-referred. In the UK, patients were recruited through various NHS sites (*n* = 119), self-referral or eating disorder charities (*n* = 96), and universities (*n* = 9).

Table 1 presents sociodemographic and clinical characteristics of the sample at baseline. On average, participants were in their early 30s and were highly educated (47.8% had a university degree). About half of the patients were in a relationship or married, and two-thirds were employed. More than 50% of the patients had received psychotherapeutic treatment for a mental health disorder in the past.

**Table 1:** Sociodemographic, clinical sample characteristics, core eating disorder symptoms and diagnoses at baseline (*N* = 337)

	All	(*N* = 337)	Intervention group	(*n* = 170)	Control group	(*n* = 167)	
	*n*	%	*n*	%	*n*	%	*P*-value^a^
In a relationship	176	52.2	88	51.8	88	52.7	0.864
Higher education^b^	274	81.3	143	84.1	131	78.4	0.182
University degree	161	47.8	83	48.8	78	46.7	0.697
In education	95	28.2	54	31.8	41	24.6	0.141
Employed^c^	215	63.8	106	62.4	109	65.3	0.578
Past treatment	194	57.6	99	58.2	95	56.9	0.802
	Mean	s.d.	Mean	s.d.	Mean	s.d.	*P-*value^d^
Age	32.10	10.18	32.09	10.04	32.11	10.35	0.982
BMI (kg/m^2^)^e^	29.38	10.05	28.97	9.56	29.80	10.54	0.450
WCS	79.32	16.06	79.53	16.50	79.10	15.65	0.807
EDE-Q total	3.88	0.93	3.87	1.00	3.89	0.86	0.898
IES	1.98	0.50	1.98	0.53	1.98	0.46	0.924
PHQ-9	15.78	6.18	15.62	6.38	15.94	5.99	0.633
GAD-7	11.86	5.74	11.83	5.76	11.89	5.73	0.920
AUDIT-C	2.73	2.57	2.79	2.55	2.66	2.60	0.629
RSE	25.29	2.34	25.39	2.36	25.19	2.32	0.428
AQoL-8D	56.83	13.93	56.80	14.61	56.86	13.25	0.968
	*n*	%	*n*	%	*n*	%	*P-*value^a^
Objective binge eating episodes	299	88.7	149	87.6	150	89.8	0.528
Vomiting	146	43.3	73	42.9	73	43.7	0.886
Medication^f^	71	21.1	37	21.8	34	20.4	0.752
Fasting	167	49.6	88	51.8	79	47.3	0.413
Excessive exercise	107	31.8	59	34.7	48	28.7	0.240
Diagnoses							0.638^g^
BED	170	50.4	83	48.8	87	52.1	
Bulimia nervosa	108	32	54	31.8	54	32.3	
OSFED	59	17.5	33	19.4	26	15.6	

BMI, body mass index; WCS, Weight Concern Scale; EDE-Q, Eating Disorder Examination-Questionnaire; IES, Intuitive Eating Scale; PHQ-9, Patient Health Questionnaire-9; GAD-7, Generalised Anxiety Disorder-7; AUDIT-C, Alcohol Use Disorders Identification Test-Consumption; RSE, Rosenberg Self-Esteem Scale; AQoL-8D, Assessment of Quality of Life-8D; BED, binge eating disorder, OSFED, other specified feeding and eating disorders.

a. *χ*²-test.

b. Educational level ≥A levels.

c. Employed includes full time, part time and self-employed.

d. *t*-test.

e. *N*_BMI_ = 334.

f. Laxatives, diuretics and/or appetite suppressants.

g. Diagnoses were tested in a 3 × 2 cross table by *χ*²-test.

The average BMI was in the overweight range (mean 29.38 kg/m^2^, s.d. = 10.05). Patients had elevated scores in eating disorder-related measures such as the EDE-Q and WCS, and reported moderately severe depressive and moderate anxiety symptoms. There were no significant differences in baseline variables between the intervention group and the control group.

Most patients (*n* = 299, 88.7%) reported regular (i.e. at least once per week) binge eating episodes, fewer than half reported regular vomiting and fasting as compensatory behaviours, and between a fifth and a third used medication or excessive exercise as means of weight control. Half of the patients fulfilled criteria for BED, about a third fulfilled criteria for bulimia nervosa and the rest fulfilled criteria for OSFED. There was no significant difference in the distribution of symptoms or diagnoses between the two study arms.

### Study drop-out/assessment completion

A total of 188 patients (55.8%; intervention group 51.8% *v.* control group 59.9%) completed at least one additional assessment after baseline. Completion rates were significantly higher in the control group compared with the intervention group at 6-month follow-up (45.5% *v.* 32.9%; *χ*² = 5.59; *P* = 0.018) and 12-month follow-up (46.1% *v.* 35.3%; *χ*² = 4.08; *P* = 0.043). Patients who dropped out after baseline (*n* = 149, 44.2%; intervention group: *n* = 82, 48.2%; control group: *n* = 67, 40.1%) reported higher baseline scores in the WCS (81.38 *v.* 77.69; *P* = 0.033) and GAD-7 (12.66 *v.* 11.22; *P* = 0.022) compared with those who did not drop out. Sociodemographic variables, prior psychotherapy and study centre did not predict drop-out. Neither the presence nor the frequency of binge eating and compensatory behaviours differed between those who did and did not drop out.

### Intervention effects

#### Primary outcome

**Fig. 2:**
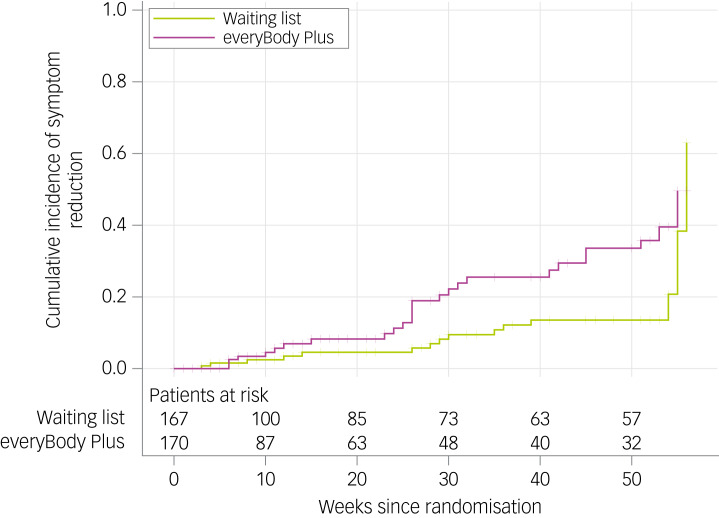
Cumulative incidence for symptom reduction in the intervention and control group. Inverse Kaplan–Meier estimator and numbers at risk shown.

Data from patients who provided any data (diary, assessment) after baseline were included in the primary analysis (*n* = 337). The cumulative incidence for symptom reduction increased significantly over time in both study arms (see Fig. 2 and Supplementary Table 4 available at https://doi.org/10.1192/bjo.2023.629, respectively). A total of 26 symptom improvement events were identified in the intervention group and 18 in the control group. Additionally, the intervention group showed a significantly more rapid symptom reduction compared with the control group (log-rank *χ*² = 5.3397; *P* = 0.021). The corresponding hazard ratio was 1.997 (95% CI 1.09–3.65; *P* = 0.025). No evidence against the proportionality assumption was detected (*P* = 0.2780).

At 6-month follow-up, the probability of being free from core eating disorder symptoms was 18.3% in the intervention group and 5.7% in the control group. At 12-month follow-up, the estimated probability increased up to 35.7% for the intervention group and 13.5% (20.7% at 54 weeks) for the control group.

Estimated number needed to treat resulted in 18.65 patients (95% CI 7.36–201.3) at 6-month follow-up and 8.59 patients at 12-month follow-up (95% CI 3.62–89.15) to be treated to induce improvement.

#### Secondary outcomes

The results of the completer analysis of secondary outcomes are described in Supplementary Table 1.

MMEM revealed a significant group×time interaction at any assessment point for eating disorder-related outcome measures (WCS, EDE-Q total, IES), anxiety (GAD-7) and quality of life (AQoL-8D), but not for depression (PHQ-9), at 12-month follow-up. For all secondary outcomes with a significant group×time interaction, intervention group patients showed larger improvements (i.e. a larger reduction in the WCS, EDE-Q, PHQ-9 and GAD-7 scores, and a larger increase in IES and AQoL-8D scores) compared with that of the control group, with small to large effect sizes (*d* = 0.29–0.82) (see Table 2).

**Table 2:** Changes in eating disorder and associated pathology between baseline and 12-month follow-up (intention-to-treat analyses, mixed model; *N* = 337)

	Baseline	Post	Effect pre–post (between)		FU6	Effect pre-FU6 (between)		FU12	Effect pre-FU12 (between)	
	Mean (s.d.)	Mean (s.e.)	*d* (95% CI)	*P*-value	Mean (s.e.)	*d* (95% CI)	*P*-value	Mean (s.e.)	*d* (95% CI)	*P*-value
BMI^a^										
Intervention group	28.97 (9.56)	29.45 (0.89)	−0.02 (−0.23 to 0.20)	0.791	28.51 (1.10)	0.12 (−0.10 to 0.33)	0.250	29.97 (0.77)	−0.10 (−0.31 to 0.11)	0.912
Control group	29.80 (10.54)	30.12 (0.88)			30.52 (1.03)			29.80 (0.78)		
WCS										
Intervention group	79.53 (16.50)	68.46 (1.57)	0.66 (0.44–0.88)	<0.001	66.72 (2.19)	0.51 (0.29–0.72)	0.002	60.50 (2.39)	0.64 (0.43–0.86)	0.001
Control group	79.10 (15.65)	78.64 (1.51)			74.45 (1.94)			70.46 (2.16)		
EDE-Q^b^										
Intervention group	3.87 (1.00)	2.79 (0.10)	0.82 (0.60–1.05)	<0.001	2.73 (0.13)	0.72 (0.50–0.94)	<0.001	2.43 (0.14)	0.67 (0.45–0.89)	<0.001
Control group	3.89 (0.86)	3.58 (0.10)			3.42 (0.12)			3.08 (0.13)		
IES										
Intervention group	1.98 (0.53)	2.43 (0.06)	−0.80 (−1.03 to −0.58)	<0.001	2.52 (0.08)	−0.82 (−1.05 to −0.60)	<0.001	2.62 (0.08)	−0.88 (−1.11 to −0.66)	<0.001
Control group	1.98 (0.46)	2.03 (0.05)			2.11 (0.07)			2.18 (0.07)		
PHQ-9										
Intervention group	15.62 (6.22)	12.24 (0.66)	0.29 (0.08–0.51)	0.020	11.53 (0.84)	0.41 (0.20–0.63)	0.016	10.01 (0.88)	0.34 (0.13–0.56)	0.060
Control group	15.94 (6.06)	14.37 (0.63)			14.38 (0.74)			12.45 (0.79)		
GAD-7										
Intervention group	11.83 (5.61)	9.63 (0.56)	0.32 (0.10–0.53)	0.007	8.89 (0.68)	0.44 (0.22–0.66)	0.005	8.16 (0.69)	0.36 (0.14–0.57)	0.027
Control group	11.89 (5.66)	11.47 (0.54)			11.44 (0.60)			10.25 (0.62)		
AUDIT-C										
Intervention group	2.79 (2.16)	2.54 (0.22)	0.10 (−0.12 to 0.31)	0.216	2.73 (0.25)	0.07 (−0.14 to 0.29)	0.479	2.62 (0.25)	0.00 (−0.21 to 0.22)	0.969
Control group	2.66 (2.83)	2.65 (0.22)			2.78 (0.23)			2.50 (0.23)		
RSE										
Intervention group	25.39 (2.26)	25.43 (0.23)	0.00 (−0.21 to 0.22)	0.955	25.07 (0.28)	0.22 (0.00–0.43)	0.201	25.53 (0.23)	0.04 (−0.18 to 0.25)	0.810
Control group	25.19 (2.12)	25.24 (0.21)			25.35 (0.24)			25.41 (0.20)		
AQoL-8D^c^										
Intervention group	56.08 (14.49)	60.97 (1.40)	−0.38 (−0.60 to −0.17)	0.001	60.64 (1.67)	−0.33 (−0.54 to −0.11)	0.043	63.96 (1.61)	−0.32 (−0.53 to −0.10)	0.039
Control group	56.86 (13.23)	56.45 (1.36)			56.89 (1.51)			60.33 (1.49)		

FU6, 6-month follow-up; FU12, 12-month follow-up; BMI, body mass index; WCS, Weight Concern Scale; EDE-Q, Eating Disorder Examination-Questionnaire; IES, Intuitive Eating Scale; PHQ-9, Patient Health Questionnaire-9; GAD-7, Generalised Anxiety Disorder-7; AUDIT-C, Alcohol Use Disorders Identification Test-Consumption; RSE, Rosenberg Self-Esteem Scale; AQoL-8D, Assessment of Quality of Life-8D.

a. *N*_BMI_ = 334.

b. EDE-Q total score.

c. We used the psychometric measures and not the utility measure here.

Noticeably, more intervention group patients stopped objective bingeing after 6 months (10/55; 18.2%) compared with the control group (3/75; 4%), and fewer patients started bingeing (non-binge eater at baseline) in the intervention group (0/55) compared with the control group (5/75; 6.7%; *P* = 0.003) (Supplementary Table 2). Furthermore, fewer objective binge eating episodes at post-intervention were observed among patients, who still reported to be symptomatic, in the intervention group (median ten episodes per 4 weeks, interquartile range (IQR): 3.5–19) compared with the control group (median 12.5 episodes per 4 weeks, IQR: 6–26; *P* = 0.047). This effect was maintained at 6-month follow-up (intervention group: 6 (IQR: 2.5–12) versus control group: 15 (IQR: 7.5–21); *P* < 0.0001) (Supplementary Table 3). The difference was also observed for fasting at post-intervention (intervention group: 0 [0–6.5] versus control group: 5 [2–12]; *P* < 0.0112) and at 6-month follow-up (intervention group: 0 [0–6] versus control group: 7.5 [0–15]; *P* = 0.008). No significant differences were identified for vomiting and laxative use.

Although the completer and ITT analyses revealed comparable effects for the eating disorder-specific outcomes (WCS, EDE-Q, IES), the two analyses differed in the results for depression, anxiety and quality of life, i.e. the completer analysis did not show a significant interaction at one or more assessment points (see Supplementary Table 1).

### Face-to-face treatment onset and utilisation

Face-to-face therapy onset was extracted from weekly diary data (available from *n* = 312/337; intervention group: *n* = 162; control group: *n* = 150; see sensitivity analyses in Supplementary Figs 2–4 and Supplementary Table 4 for further methodology). Onset was defined as first diary entry (after baseline) that reported at least one therapy session during the past week. In the intervention group, 72 (44.4%) patients reported therapy onset, whereas in the control group, 75 (50%) patients reported onset during the study (*P* = 0.2971; *n* = 147). Mean onset time was 10.5 weeks (s.d. = 10.7, range: 0.14–55) in the intervention group and 13.9 weeks (s.d. *n* = 15.0, range = 0.14–53.6) in the control group (*P* = 0.6157). Including therapy onset as a time-dependent covariable in the Cox model confirmed our primary result, i.e. the everyBody Plus intervention increased the chance for early symptom improvement events (*P* = 0.0281; hazard ratio 1.967, 95% CI 1.076–3.596) (Supplementary Table 5). The analysis of the same model with an additional interaction term between randomised group and therapy onset yielded similar results for the main effects (Supplementary Table 6). The interaction term did not indicate any differential effect within the randomised groups with respect to therapy onset.

### Adherence, satisfaction and working alliance

#### Adherence

The 170 patients in the intervention group completed an average of five sessions (mean 4.79, s.d. = 3.01). Sixty-seven patients (39.4%) completed the full course of the intervention and 95 patients (55.8%) completed at least half of the intervention content (Supplementary Fig. 1). Thirteen patients (7.6%) completed none of the sessions. All patients logged on to the study platform after baseline assessment at least once.

A total of 167 patients in the intervention group (98.2%) provided at least one symptom diary (mean 18.4, s.d. = 19.3, range: 1–61), which corresponds to 35.4% of the recommended symptom diary entries. A total of 112 patients (64.1%) used the self-reflection diary at least once (mean 7.21, s.d. = 11.29, range: 1–95). The group discussions were used by 76 patients (44.7%), who posted on average 6.07 messages (s.d. = 6.62, range: 1–37), and 136 patients (80.0%) wrote at least one message to their online coach (mean 6.43, s.d. = 6.00, range: 1–30).

In the control group, 156 patients (93.4%) made at least one entry in the symptom diary (mean 24.1, s.d. = 21.0).

#### Session ratings

The individual sessions were rated as good on a scale from 0 to 4 (mean 2.95, s.d. = 0.53, range: 2.72 (session 8) to 3.12 (session 4)).

#### Working alliance

intervention group patients showed high alliance scores on each scale of the WAI-SR: Task (mean 3.16, s.d. = 0.96), Goal (mean 3.67, s.d. = 0.92) and Bond (mean 4.38, s.d. = 1.03). On average, patients agreed between ‘fairly often’ and ‘very often’ with the tasks and goals of the intervention, and between ‘very often’ and ‘always’ with the quality of the interpersonal bond with the online coach.

## Discussion

The current RCT represents the first study to assess the effectiveness of a web-based guided self-help intervention for patients with bulimic-spectrum eating disorder, awaiting face-to-face out-patient treatment in Germany and the UK. We also aimed to assess acceptance of such an intervention to bridge the waiting time for out-patient treatment in two European countries with different healthcare systems. Overall, results were encouraging. Although the time to face-to-face therapy onset was comparable across the two conditions, at 12-month follow-up, almost three times as many patients in the intervention group achieved abstinence of core eating disorder symptoms over a period of at least 4 weeks, compared with the control group (35.7% *v.* 13.5%). Patients in the intervention group also showed a significantly more rapid symptom reduction over time compared with the control group. The more significant improvements were not only eating disorder-related symptoms (e.g. weight, shape and eating concerns, restrictive and intuitive eating), but also general psychopathology (e.g. anxiety, depression and quality of life) over a year.

To our knowledge, no trial has been conducted in which a web-based guided self-help intervention for eating disorders was explicitly offered to bridge waiting times for out-patient face-to-face treatment. The meta-analysis by Linardon et al^3^ included eight treatment-focused studies that yielded positive effects on eating disorder psychopathology (e.g. weight and shape concerns, dietary restraint). However, the frequency and abstinence of binge eating were not included and the studies did not intend to bridge waiting time. The current study demonstrates that the everyBody Plus intervention not only benefits females with subclinical level of eating disorders,^12^ but it also leads to significant improvement among females with clinical threshold of eating disorders in routine clinical and pragmatic settings. The intervention was well-accepted and working alliance ratings with the online therapist were high, and at least comparable to scores found in psychotherapy patients.^25^

### Strengths and limitations

The current study has several strengths. No other trial has so far addressed the potential of implementing a web-based intervention into routine care. The high therapeutic alliance ratings, especially the high quality of the interpersonal bond to the online coach that the participants had never met, underline this potential and the credibility of the everyBody Plus intervention. The recruitment of a large clinical sample, mostly referred to the trial through clinical services or therapists in private practices, is rather unique. The primary outcome was selected to reflect a highly clinically relevant criterion, i.e. abstinence of core symptoms over at least 4 weeks. This criterion is more difficult to achieve for patients and also rarely reported in web-based or other guided self-help trials.^3^ The study was well powered, with patients being recruited from two different countries and health services, which increases the generalisability of the results.

However, the strength of a multi-country trial also presented with some challenges. Healthcare systems between Germany and the UK are not completely comparable, and treatment provision differs. Although patients in the UK were mostly recruited through specialised eating disorder services, such services hardly exist in Germany and the recruitment strategy therefore focused on recruiting through private practices and university out-patient services. Accordingly, recruitment rates were lower in Germany compared with the UK. Subsequently, a self-recruitment strategy was introduced during the trial, indicating a need for further information for therapists in routine care on the effectiveness of web-based interventions for eating disorder. In addition, the study highlights the need for improving the German mental health services covered by statutory health insurances. Unfortunately, our study was conducted before legal changes in Germany were implemented: since 2020, evidence-based online interventions for specific mental health conditions can now be registered as medical products prescribed by therapists, and subsequently reimbursed by statutory health insurance companies. Because recruitment ended in 2019, this potential ‘door opener’ could not be utilised. In the UK, however, guided self-help interventions are recommended by the National Institute for Health and Care Excellence guidelines for eating disorders as a first step in the treatment of bulimic-spectrum disorders,^4^ and specialist NHS services, which often struggle to treat patients in a timely manner and typically have lengthy waiting lists, welcome their use.

A further limitation was the high drop-out rate regarding the post and follow-up assessments (twice as high in the current trial at post-intervention compared with mean drop-out in other web-based trials for eating disorder: 50% *v.* 25.3%).^3^ However, this was not unexpected in the context of bridging the waiting time rather than using the intervention as a stand-alone intervention: between 45.4 and 50% of the patients started treatment on average 10 weeks after randomisation. Face-to-face treatment uptake might have affected adherence to the everyBody Plus intervention and study assessments. Importantly, irrespective of face-to-face therapy uptake, the intervention led to faster improvements in eating disorder symptoms. Possible subsequent deteriorations after initially being counted as abstinent from core eating disorder symptoms were not included in the definition of the primary outcome. However, the main objective of the intervention was to shorten time to improvement when waiting for treatment. Subsequent changes in eating disorder symptoms will be largely related to face-to-face therapy.

The generalisability of the results could also be limited because of the participant characteristics. Considering the significantly higher prevalence of eating disorders in females compared with males,^4^ the everyBody Plus intervention was female-centred and participation in the study was restricted to female patients. Also, we did not collect ethnicity data. In addition, the mean BMI of the sample was in the overweight range and the results may not be representative for bulimia nervosa patients in the normal weight range. In line with this, qualitative feedback from some UK patients indicates a potential need for adaptation of the intervention for patients with larger bodies.^26^

### Clinical and research implications

The results of our study show that public health systems could better utilise evidence-based, web-based guided self-help to bridge waiting times and thus facilitate the improvement of core clinical outcomes for patients with eating disorders. As patients received basic psychoeducational information about eating disorders in everyBody Plus, this can save time during subsequent face-to-face treatment that can be used to address more persistent eating disorder symptoms (e.g. vomiting) or other related problems (e.g. interpersonal and emotional problems, comorbidities). Also, engaging with the web-based intervention during the waiting period could facilitate and/or sustain motivation for change, which is a crucial component in eating disorder treatment. A better understanding of the eating disorder, treatment motivation and first symptom improvements can result in fewer face-to-face sessions subsequently needed, which in turn saves resources and makes the combination of web-based intervention and subsequent treatment a more cost-effective alternative to pure face-to-face treatment.

Among those who were still symptomatic at post-intervention, fewer binge eating and fasting episodes were found in the intervention group versus control group. However, there was no difference in vomiting and laxative use. This preliminary finding may highlight a need for clinicians to specifically attend to the compensatory behaviours in face-to-face sessions, since these may be the more persistent and require more intensive input. Providing conditional and tailored content for those patients who report vomiting and laxative use may be indicated; however, it is unclear whether expanding the content will result in a significant difference in the frequency. Future research should examine the characteristics of patients who may benefit more from this form of treatment, to ensure timely access to treatment and help with waiting list management. In addition, we expect the planned moderator, sensitivity and health–economic analyses^27,28^ to provide indicators of predictors of intervention effects, drop-out, site differences and cost–benefit ratios.

Overall, the everyBody Plus intervention has demonstrated potential to be provided in routine care settings for bulimic-spectrum eating disorder in both Germany and the UK. The intervention could thus help bridge the treatment gap for patients with eating disorder, lead to faster and greater reductions in core eating disorder symptoms, and reduce burden and cost.

## Supporting information

Vollert et al. supplementary material Vollert et al. supplementary material

## Data Availability

Anonymised data that support the findings of this study are available from the corresponding author, B.V., upon reasonable request.
