# Supplementary material for: Using web-based, guided self-help to bridge the waiting time for face-to-face out-patient treatment for bulimic-spectrum disorders: randomised controlled trial
Source: BJPsych Open. 2024 Feb 26;10(2):e53. doi: 10.1192/bjo.2023.629 (PMC10897701; doi:10.1192/bjo.2023.629)
Supplement: Vollert et al. supplementary material [file S2056472423006294sup001.pdf]

Supplementary Material

of the paper

Using web-based, guided self-help to bridge waiting time for face-to-face outpatient treatment for bulimic-spectrum disorders: A randomised controlled trial

by Vollert et al.

Table of contents

Supplemental Tables 2

Table S1. Secondary outcomes at baseline, post and follow-up assessments for patients who completed the respective assessment. 2

Table S2. Change in ED core symptoms between baseline and FU6. 3

Table S3. Difference in the frequency of ED core symptom in patients who reported the respective symptom at the assessment points. 4

Supplemental figure 5

Fig. S1. Session completion rates of IG participants. 5

Sensitivity analyses of the primary outcome adjusted for F2F therapy onset 6

Fig. S2. All diary entries are displayed over time indicating therapy sessions by colour. 6

Table S4. Numbers at risk for the primary analysis and the sensitivity analysis censoring at therapy onset. 7

Fig. S3. Event and censoring times vs time to therapy onset for the N=147 (IG: 72; CG: 75) patients who reported onset. 7

Fig. S4. Kaplan-Meier estimates (inverse) after censoring all patients (censored or with event) with therapy sessions reported at their onset time. 7

Table S5. Time-dependent Cox proportional hazard model with treatment onset as time dependent variable. 8

Table S6. Time-dependent Cox proportional hazard model including study arm + time-dependent treatment onset + interaction. 8

Supplemental Tables

Table S1. Secondary outcomes at baseline, post and follow-up assessments for patients who completed the respective assessment.

		**Baseline**		**Post**					**FU6**				**FU12**			
		**N**	**mean**	**SD**	**N**	**mean**	**SD**	**Cohen's *d***	**N**	**mean**	**SD**	**Cohen's *d***	**N**	**mean**	**SD**	**Cohen's *d***
**BMI**	IG	169	28.97	9.56	78	28.52	10.46	-0.02	56	27.57	8.75	0.11	60	28.06	10.16	0.00
	CG	165	29.80	10.54	91	30.06	10.97	p=.790	76	31.11	13.88	p=.251	77	30.43	11.28	p=.953
**WCS**	IG	170	79.53	16.50	82	66.65	19.23	**0.60**	56	64.58	22.58	**0.44**	62	58.98	22.58	**0.59**
	CG	167	79.10	15.65	94	78.32	15.11	**p<.001**	77	72.97	18.36	**p=.004**	78	68.18	20.07	**p=.001**
**EDE-Q**	IG	170	3.87	1.00	82	2.82	1.22	**0.83**	56	2.61	1.25	**0.67**	62	2.32	1.28	**0.65**
	CG	167	3.89	0.86	92	3.62	0.89	**p<.001**	77	3.40	1.12	**p<.001**	78	3.03	1.21	**p<.001**
**IES**	IG	170	1.98	0.53	80	2.44	0.62	**-0.82**	56	2.53	0.70	**-0.78**	61	2.64	0.70	**-0.87**
	CG	167	1.98	0.46	92	2.02	0.52	**p<001**	76	2.13	0.56	**p<.001**	77	2.24	0.67	**p<.001**
**PHQ-9**	IG	170	15.62	6.38	79	11.56	6.83	0.25	56	10.48	6.88	0.35	60	9.40	7.31	0.32
	CG	167	15.94	5.99	91	14.43	6.63	p=.050	76	14.33	7.28	p=.050	77	12.30	7.55	p=.101
**GAD-7**	IG	170	11.83	5.76	79	9.08	5.52	**0.30**	56	8.02	5.62	**0.34**	60	7.47	5.79	0.31
	CG	167	11.89	5.73	91	11.16	6.10	**p.012**	76	11.32	5.92	**p=.038**	77	9.86	6.07	p=.070
**AUDIT-C**	IG	170	2.79	2.55	79	2.22	2.07	0.06	56	2.04	2.10	0.03	60	2.15	1.88	-0.08
	CG	167	2.66	2.60	90	2.77	2.82	p=.407	76	2.58	2.71	p=.741	77	2.49	2.59	p=.527
**RSE**	IG	170	25.39	2.36	79	25.46	2.01	0.03	56	25.07	2.22	0.28	60	25.60	1.62	0.17
	CG	167	25.19	2.32	90	25.23	2.21	p=.848	76	25.21	2.25	p=.117	77	25.36	1.94	p=.356
**AQoL-8D**	IG	170	56.80	14.61	79	63.05	15.49	**-0.31**	56	63.26	15.63	-0.25	60	66.32	14.41	-0.25
	CG	167	56.86	13.25	90	56.08	15.42	**p=.002**	76	56.61	15.21	p=.083	77	60.27	15.84	p=.066

Notes. IG=Intervention group; CG=control group; SD=standard deviation; BMI=Body Mass Index; WCS=Weight Concern Scale; EDE-Q=Eating Disorder Examination-Questionnaire; IES=Intuitive Eating Scale; PHQ-9=Patient Health Questionnaire-9; GAD-7=Generalized Anxiety Disorder-7; AUDIT-C=The Alcohol Use Disorders Identification Test-Consumption; RSE=Rosenberg Self-Esteem Scale; AQoL-8D=Assessment of Quality of Life-8D

In a completer analysis of secondary outcomes, we found noticeable changes between baseline and post-assessments for WCS, EDEQ, IES, GAD7 and AQoL-8D. At 6-month FU WCS, EDE-Q, IES and GAD-7 were still noticeably different between IG and CG. At the 12-month FU only WCS, EDE-Q and IES were found to differ in the completer analysis.

Table S2. Change in ED core symptoms between baseline and FU6.

	N		Patients with improvement at FU6		Patients who showed an onset at FU6		*p*-value	Patients with improvement at FU6		Patients who showed an onset at FU6		*p*-value
	IG	CG	IG	CG	IG	CG		IG	CG	IG	CG	
			Objective binges					Vomiting				
Baseline-Post	81	92	8 (9.9%)	7 (7.6%)	2 (2.5%)	7 (7.6)	0.3269	3 (3.7%)	5 (5.4%)	0 (0%)	3 (3.3%)	0.2811
Baseline-FU6	55	75	**10 (18.2%)**	**3 (4%)**	**0 (0%)**	**5 (6.7%)**	**0.0032**	5 (9.1%)	5 (6.7%)	1 (1.8%)	4 (5.3%)	0.5817
Baseline-FU12	61	77	13 (21.3%)	9 (11.7%)	1 (1.6%)	2 (2.6%)	0.3528	11 (18.0%)	9 (11.7%)	2 (3.3%)	4 (5.2)	0.5690

Notes. IG=Intervention group; CG=control group

The Number needed to treat (NNT) for the noticeable difference between IG and CG at FU6 is NNT: 1/(0.182-0.04)=7.04

Table S3. Difference in the frequency of ED core symptom in patients who reported the respective symptom at the assessment points.

	**Baseline**				**Post**				**FU6**				**FU12**			
	**N**		**Median (Q1,Q3)**		**N**		**Median (Q1,Q3)**		**N**		**Median (Q1,Q3)**		**N**		**Median (Q1,Q3)**	
	**IG**	**CG**	**IG**	**CG**	**IG**	**CG**	**IG**	**CG**	**IG**	**CG**	**IG**	**CG**	**IG**	**CG**	**IG**	**CG**
Frequency of …																
objective binges	145	146	15 (8,20)	16 (10,25) p=0.1221	**72**	**78**	**10 (3.5,19)**	**12.5 (6,26) p=0.0495**	**52**	**64**	**6 (2.5,12)**	**15 (7.5,21) p<.0001**	57	62	7 (2,14)	8 (3,20) p=0.1878
vomiting	67	70	15 (4,28)	14 (4,28) p=0.8596	30	44	12 (4,20)	10 (3,22.5) p=0.9956	24	30	4.5 (1.5,10)	7.5 (2,25) p=0.1542	25	32	2 (0,12)	4.5 (0.5,18) p=0.2218
laxative intake	22	22	15 (3,18)	7 (2,18) p=0.1708	10	10	4 (0,28)	5.5 (0,17) p=0.9377	7	6	0 (0,28)	4.5 (0,8) p=0.7634	9	8	0 (0,8)	3 (1,11.5) p=0.6532
fasting	84	77	10 (5,15.5)	6 (4,13) p=0.1144	**44**	**41**	**0 (0,6.5)**	**5 (2,12) p=0.0119**	**31**	**32**	**0 (0,6)**	**7.5 (0,15) p=0.0084**	36	35	0 (0,7.5)	5 (0,8) p=0.2616

Notes. IG=Intervention group; CG=control group; P-values are Mann Whitney-U test results; Frequencies represent the number of occurrences within the last 4 weeks.

Supplemental figure

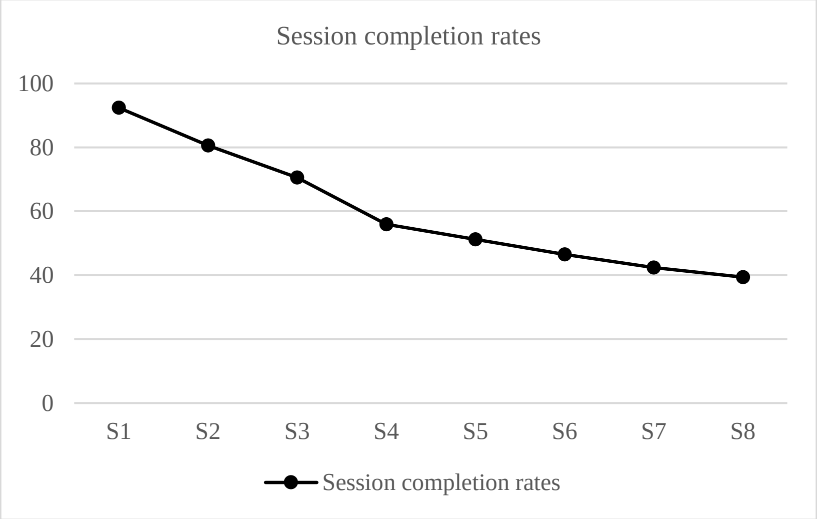

Fig. S1. Session completion rates of IG participants.

Sensitivity analyses of the primary outcome adjusted for F2F therapy onset

We analysed the diary data for reported therapy onset after day 0 (i.e. day 0 diaries were excluded from this analysis). Participants could indicate this by answering the following question in the symptom diary:

> DE: “Falls Sie bereits mit einer Psychotherapie begonnen haben: Wie viele Therapiestunden haben Sie in der letzten Woche absolviert?”
> UK: “If you have already started face-to-face therapy, how many sessions have you attended in the last week?”

Fig. S2 shows the distribution of diaries over time, indicating reported therapy sessions by color.

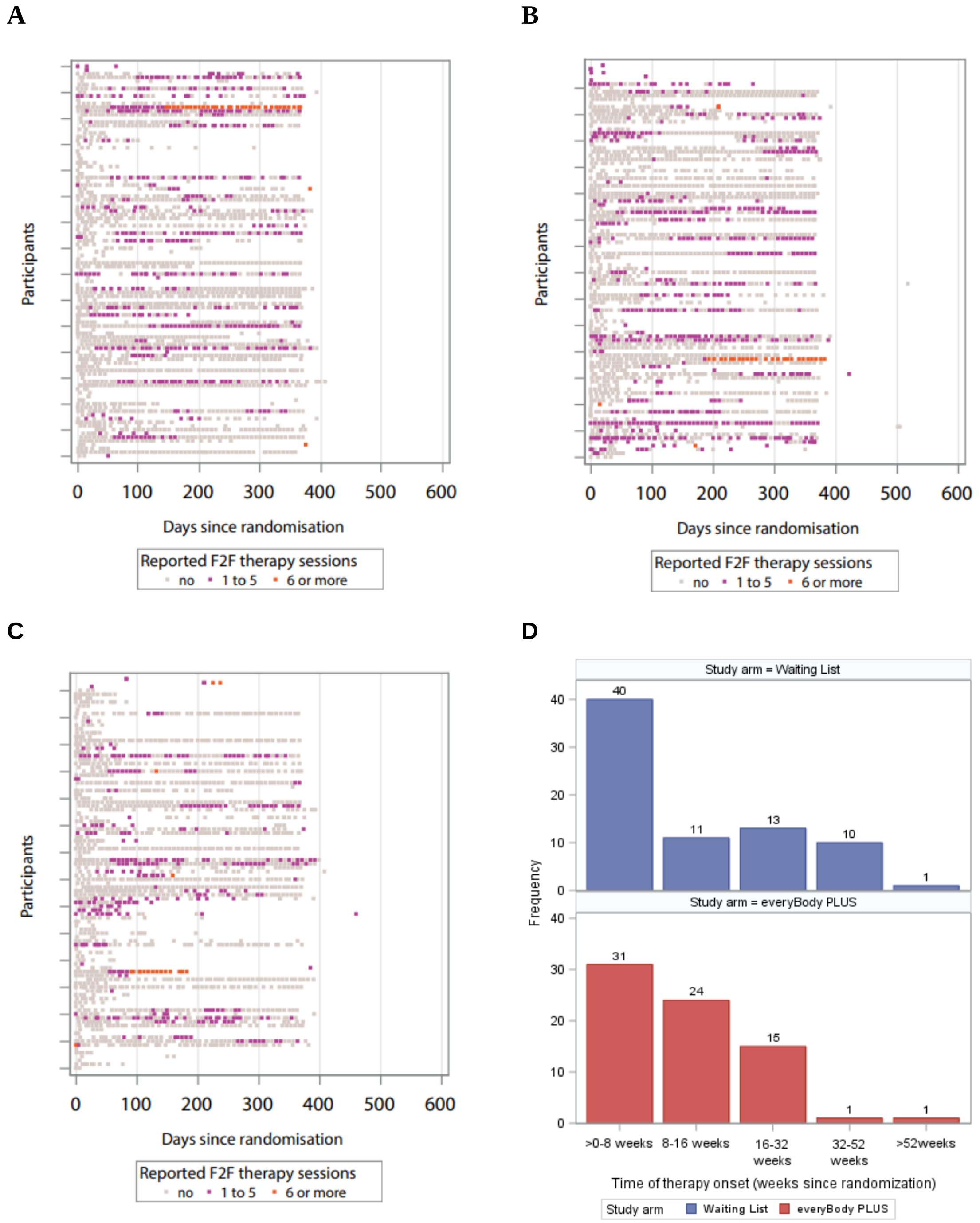

Fig. S2. All diary entries are displayed over time indicating therapy sessions by colour.
N=312 participants (y-axis) were distributed among three panels A-C, for visibility. Panel D shows the extracted therapy onset times in broader categories for N=147 patients with an onset.

Finally N=312 patients were analysable with regard to therapy onset. N=25 patients did not provide diaries or only at day 0. N=147 (47.12%) patients reported an onset.

<u>Sensitivity analysis: Censoring at therapy onset</u>

For this sensitivity analysis we censored for therapy onset in the time-to-event data. By this censoring times of participants shift to earlier time points and, more importantly, symptom improvement events that occur after therapy onset are now included as censored observations. This reduces the power of the analysis. **Table S4** shows the comparison in the number of events and the numbers at risk between the primary analysis as shown in the main text and this sensitivity analysis.

Table S4. Numbers at risk for the primary analysis and the sensitivity analysis censoring at therapy onset.

	**Primary analysis**						**Sensitivity analysis (censoring at therapy onset)**					
**Time in weeks**	0	10	20	30	40	Max	0	10	20	30	40	Max
	**Numbers at risk**						**Numbers at risk**					
**CG**	167	100	85	73	63	57	167	66	46	35	28	20
**IG**	170	87	63	48	40	32	170	61	32	20	17	14
	**Cumulative SI event numbers**						**Cumulative SI event numbers**					
**CG**	0	3	5	9	12	18	0	3	4	4	5	6
**IG**	0	5	8	17	19	26	0	5	5	8	8	11

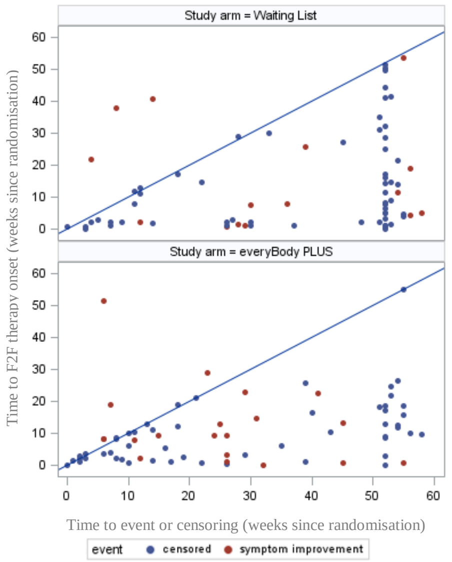

Fig. S3. Event and censoring times vs time to therapy onset for the N=147 (IG: 72; CG: 75) patients who reported onset.
If therapy onset is before censoring/event time (lower triangle), the therapy onset time will be used in this sensitivity analysis as censoring time.

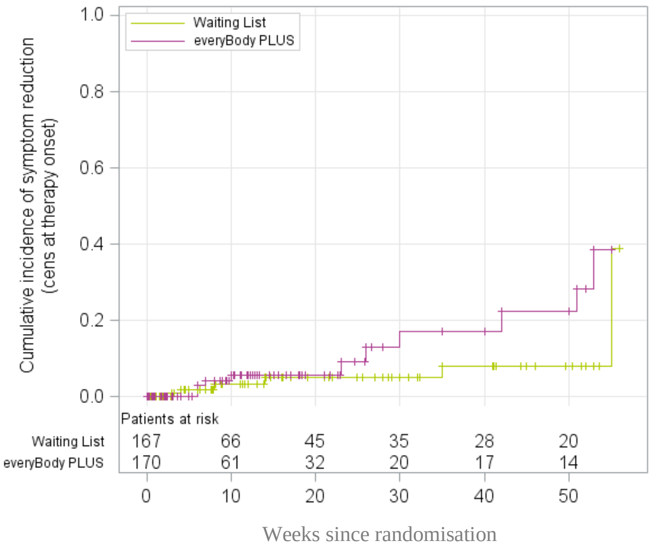

Fig. S4. Kaplan-Meier estimates (inverse) after censoring all patients (censored or with event) with therapy sessions reported at their onset time.
Log-Rank test result: Chi-Squared: 2.5638, p=0.1093.

Sensitivity analysis: Time-dependent Cox regression

We performed a better analysis (without power loss) by applying a Cox proportional hazard model with therapy onset as a time-dependent variable. Time dependence was introduced by indicator variables for therapy onset at discrete weeks (1-56). All weeks after the onset were set to 1 (i.e. therapy started). **Table S5** shows the final Cox model coefficients.

Table S5. Time-dependent Cox proportional hazard model with treatment onset as time dependent variable.

Factor	DF	Parameter Estimate	Standard Error	Chi-Square	Pr > ChiSq	Hazard Ratio	95%CI	
**everyBody Plus vs. Waiting list**	1	0.67635	0.30791	4.8249	0.0281	1.967	1.076	3.596
**Time dependent variable: therapy onset**	1	0.57861	0.34275	2.8498	0.0914	1.784	0.911	3.492

The randomised group is still the main influencing factor while simultaneously time dependence could not be proven as relevant factor here.

Also, analysing therapy onset alone in a time-dependent Cox model does not allow to clearly prove an association with regard to symptom improvement (HR: 1.806, 95%CI: 0.927-3.518, p=0.0824).

Finally, we analysed an interaction model between the everyBody program and treatment onset (Y= study arm + time-dependent treatment onset + interaction). This study's power did not suffice to prove any detailed effects on the interaction in this model (**Table S6**, p>0.05) but confirmed the benefit of the everyBody program.

Table S6. Time-dependent Cox proportional hazard model including study arm + time-dependent treatment onset + interaction.

Factor	DF	Parameter Estimate	Standard Error	Chi-Square	Pr > ChiSq
**everyBody Plus vs Waiting list**	1	1.08442	0.54328	3.9842	0.0459
**Time dependent variable: therapy onset**	1	0.95016	0.53753	3.1246	0.0771
**Interaction**	1	-0.62305	0.66303	0.8830	0.3474
